# Disaster Literacy

**DOI:** 10.1093/geroni/igaa057.2430

**Published:** 2020-12-16

**Authors:** Lisa Brown, Lindsay Peterson

**Affiliations:** 1 Palo Alto University, Palo Alto, California, United States; 2 University of South Florida, Tampa, Florida, United States

## Abstract

People who plan ahead typically fare better during the response and recovery phases of a disaster. However, problems arise when the needs, wants, abilities, and resources of vulnerable people are not adequately considered. The lack of alignment between the literacy demands of existing materials and the literacy skills of many vulnerable subgroups limits their ability to understand and effectively use potentially life-saving information. Existing health literacy models that have demonstrated effectiveness in changing health behaviors and improving outcomes is a first step to reducing disaster-related morbidity and mortality in low resource and low literacy areas. This presentation will 1) describe how interdisciplinary collaborations can be used to address this public health issue, 2) explain how health literacy techniques can be applied when developing disaster materials, and 3) present research data on a social marketing campaign to improved disaster preparedness of older adults. Part of a symposium sponsored by Disasters and Older Adults Interest Group.

